# Prognostic Factors of Early Morphological Response to Treatment with Ranibizumab in Patients with Wet Age-Related Macular Degeneration

**DOI:** 10.1155/2015/867479

**Published:** 2015-03-04

**Authors:** Oldřich Chrapek, Jiří Jarkovský, Martin Šín, Jan Studnička, Petr Kolář, Barbora Jirková, Ladislav Dušek, Šárka Pitrová, Jiří Řehák

**Affiliations:** ^1^Department of Ophthalmology, Faculty of Medicine and Dentistry, Palacky University, I. P. Pavlova 6, 775 20 Olomouc, Czech Republic; ^2^Institute of Biostatistics and Analyses, Faculty of Medicine and Faculty of Science, Masaryk University, Kamenice 126/3, 625 00 Brno, Czech Republic; ^3^Department of Ophthalmology, Faculty of Medicine in Hradec Králové, Charles University in Prague and University Hospital in Hradec Králové, Sokolská 581, 500 03 Hradec Králové, Czech Republic; ^4^Department of Ophthalmology, University Hospital, Jihlavská 340/20, 625 00 Brno, Czech Republic; ^5^Private Eye Clinic, V Hůrkách 1296/10, 158 00 Prague, Czech Republic

## Abstract

*Aim*. To assess the significance of age, gender, baseline best corrected visual acuity, baseline macula thickness, and type and size of choroidal neovascularization in early morphological therapeutic response to ranibizumab treatment in patients with the wet form of age-related macular degeneration. *Methods*. From 09/2008 to 06/2013 we evaluated 1153 newly diagnosed, treatment-naïve patients treated with ranibizumab. Based on the morphological findings in the macula following the initial 3 injections of ranibizumab, the patients were divided into two groups based on active and inactive choroidal neovascularization. *Results*. After the initial 3 injections of ranibizumab, we examined the sample of 841 eyes with active CNV and 312 eyes with inactive CNV. In the inactive group, we found a statistically higher proportion of occult CNV (*P* < 0.001) and lower incidence of CNV greater than 5DA (*P* < 0.001) compared with the active group. We found no statistically significant difference in age, gender, baseline best corrected visual acuity, or baseline macula thickness between the inactive and active groups. *Conclusion*. Occult CNV and CNV smaller than 5DA are optimistic factors for a better morphological therapeutic response at the beginning of ranibizumab treatment.

## 1. Introduction

Age-related macular degeneration (AMD) is subdivided into the dry form and the wet form. According to Bressler et al., 90% of legal blindness from AMD is caused by the wet form [1]. The Minimally Classic/Occult Trial of the Anti-VEGF Antibody Ranibizumab in the Treatment of Neovascular Age-Related Macular Degeneration (MARINA) evaluated the benefit of ranibizumab in a dose of 0.5 mg per month. At 12 months, 95% of patients had lost <15 Early Treatment Diabetic Retinopathy Study (ETDRS) letters from baseline and 34% of patients had a best corrected visual acuity (BCVA) gain of ≥15 ETDRS letters [2]. The study Ranibizumab in Patients with Subfoveal Choroidal Neovascularization (CNV) Secondary to Age-Related Macular Degeneration (SUSTAIN study) was designed to further evaluate the safety, tolerability, and efficacy of optical coherence tomography (OCT)/BCVA-guided, individualized, and flexible pro re nata (PRN, as needed) dosing regimen for ranibizumab. At 12 months, 92.5% of patients had lost <15 ETDRS letters from baseline and 19.3% of patients had a BCVA gain of ≥15 ETDRS letters [3].

Whether the fixed or PRN regimen was used in the treatment of wet AMD, the therapeutic response of individual patients was not always the same. Current observations suggest that the factors which affect the response to the initial treatment with ranibizumab are individual.

The aim of this study was to assess the importance of various factors (age, gender, baseline BCVA, baseline macula thickness, and type and size of CNV) for early morphological therapeutic response to ranibizumab in clinical practice.

## 2. The Population and Methodology

The treatment of patients with wet AMD is centralized into 9 tertiary referral centres in the Czech Republic (see Appendix). Anonymised data on treatment efficacy and safety have been consecutively entered into the Czech national database AMADEuS (Age-Related Macular Degeneration in patients in the Czech Republic) since September 2008. The main aim of this registry is to collect basic epidemiologic data on patients diagnosed with wet AMD in the Czech Republic, document standard diagnostic and therapeutic patterns, and assess treatment efficacy in standard clinical practice. The data collection is independent of all treatment decisions; it does not affect a patient's access to treatment and fully complies with all ethical as well as legal requirements for noninterventional data collection in the Czech Republic. All patients have given written informed consent to the treatment, as well as data collection. The reported investigations were in accordance with the principles of the current version of the Declaration of Helsinki.

The data are recorded from the moment of diagnosis and start of treatment at regular 3-month intervals for half a year. In the following period, the record is completed every 6 months. Each record presumes biomicroscopic examination of the retina, determination of BCVA using the ETDRS chart, and an OCT examination (OCT 3 Stratus). The first visit involves fluorescein angiography (FA); indocyanine green angiography (ICGA) is used only if it is necessary for determining a diagnosis. Based on the examinations, compulsory and optional data are specified. The compulsory data always include BCVA expressed by the number of ETDRS letters, the central thickness of macula in 1 mm of macula in *μ*m, and volume in 6 mm of macula in mm^3^. The first visit also involves recording age and gender and measuring type and size of CNV using FA or ICGA. Patients with diagnosed wet form of AMD who meet the Czech Society of Ophthalmology criteria for initiation of treatment with the ranibizumab are entered into the registry. Ranibizumab therapy in the Czech Republic is indicated in patients with AMD who are older than 50 years, with predominantly classic, minimally classic, or occult CNV in subfoveal localization, a BCVA score between 70 and 35 letters (20/40–20/200 Snellen equivalent), total macular lesion area ≤8 disc area (DA), and submacular haemorrhage ≤25% of the total macular lesion area. Minimally classic and occult CNV must show signs of activity in the form of the presence of hard exudates, subretinal haemorrhages, or decrease in BCVA within the last 3 months by ≥10 letters of the ETDRS chart. In patients treated with ranibizumab in a dose of 0.5 mg, there are two separate phases: the loading phase, followed by a PRN phase. In the loading phase, patients receive 3 consecutive monthly injections of ranibizumab (months 0–2), followed by a PRN phase when further treatment is given between and including months 3 and 11 according to the retreatment criteria.

Retreatment with ranibizumab is performed if the patient's BCVA worsened against BCVA recorded in the previous visit and if there is a demonstrable macular edema on OCT examination. The PRN method of application is also followed in the second year and all succeeding years of patient treatment.

Our study assessed the influence of age, gender, baseline BCVA, baseline macula thickness, and type and size of CNV on early morphological therapeutic response to ranibizumab in clinical practice. We studied these factors in terms of anatomical changes in the macula after 3 consecutive monthly injections of ranibizumab in the loading phase (months 0–2). The monitored factors: age, gender, baseline best corrected visual acuity, baseline macula thickness, and type and size of choroidal neovascularization nor any others parameters (visual acuity, OCT) had no influence on the treatment scheme in the loading phase.

From 01/09/2008 to 24/6/2013, 1153 newly diagnosed, treatment-naïve patients treated with ranibizumab were entered into the registry.

Following the 3 initial injections of ranibizumab, the patients were divided into two groups based on the morphological findings in the macula: a group with active CNV and a group with inactive CNV. In the group with active CNV, OCT screening revealed intraretinal macular edema, subretinal fluid accumulation, retinal pigment epithelium (RPE) detachment, fibrovascular RPE detachment, or the combination of all these findings. No vitreomacular traction was revealed.

In the group with inactive CNV, OCT screening revealed restored foveal depression and a scar at the site of CNV with no signs of exudation was apparent. No intraretinal macular edema, subretinal fluid accumulation, RPE detachment, or fibrovascular RPE detachment was found.

In both groups, we assessed the following parameters: gender and age of patients, type and size of CNV, baseline BCVA on the ETDRS chart, and baseline macular thickness.

Standard descriptive statistics were applied in the analysis: absolute and relative frequencies for categorical variables and median supplemented with 5th–95th percentiles and mean supplemented by 95% confidence interval for continuous variables. The statistical significance of differences between groups was analyzed using Pearson's chi-square test for categorical variables and Mann-Whitney *U* test for continuous variables.*α* = 0.05 was adopted as the level of statistical significance in all analyses.

The analysis was computed using the software PASW Statistics 19.0.1. (SPSS, Inc. 2010) and performed by the Institute of Biostatistics and Analyses at Masaryk University, Brno, operating independently of any AMD treating centre in the Czech Republic.

## 3. Results

The sample included 1092 patients, 38.6% men, average age 73.3 years (SD: 8.4), and 61.4% women, average age 74.2 years (SD: 8.6). The analysis included 1153 treated eyes; the right eye was treated 561 times, the left eye 592 times (both eyes were treated 61 times).

After the initial 3 injections (day 0, month 1, and month 2) of ranibizumab, in month 3 we examined the sample of 1153 eyes. Of these there were 841 eyes with active CNV (the active group) and 312 eyes with inactive CNV (the inactive group).

The sample in the active group included 37.9% of men and 62.1% of women. The sample in the inactive group included 41% of men and 59% of women (*P* = 0.338, Pearson's chi-square test).

The active and the inactive group included 29.3% and 27.9% of patients at the age <70 years, 43.4% and 42.9% of patients aged 70–80 years, and 27.3% and 29.2% of patients at the age >80 years (*P* = 0.237, Mann-Whitney *U* tests), respectively.

The active and the inactive group included 31% and 20.2% of patients with predominantly classic CNV, 21% and 19.9% with minimally classic CNV, and 47.9% and 59.9% with occult CNV, respectively. The inactive group showed statistically significantly higher presence of occult membranes and statistically significant lower presence of predominantly classic CNVs compared with the active group (*P* < 0.001, Pearson's chi-square test).

The active and the inactive group included 23.8% and 26.9% of patients with CNV < 2 disc areas (DA), 66.8% and 70.2% of patients with CNV 2–5 DA, 9.4% and 2.9% of patients with CNV > 5 DA, respectively. The inactive group showed statistically significantly lower presence of CNV > 5 DA compared with the active group (*P* < 0.001, Pearson's chi-square test).

The baseline BCVA in the range of 15–30 ETDRS letters was shown in 10.2% of patients in the active group and 10.9% of patients in the inactive group. The BCVA in the range of 31–60 ETDRS letters was shown in 62.5% of patients in the active group and 55.1% of patients in the inactive group. The baseline BCVA of >60 ETDRS letters was shown in 27.2% of patients in the active group and 34% of patients in the inactive group. The median of the baseline BCVA was 54 (5–95 percentiles: 22–73) and 55 (5–95 percentiles: 23–75) in the active and inactive group, respectively. We found no statistically significant difference in the value of the baseline BCVA between the groups (*P* = 0.066, Mann-Whitney *U* test).

19.3% of patients in the active group and 15.6% of patients in the inactive group had a baseline macular thickness of <250 *μ*m. The baseline macular thickness in the range of 250–400 *μ*m was shown in 51.7% of patients and 58.5% of patients, respectively, and baseline macular thickness of >400 *μ*m was shown in 29% and 25.9% of patients, respectively. The median of the baseline macular thickness was 330 *μ*m (5–95 percentiles: 190–600) and 337 *μ*m (5–95 percentiles: 201–535) in the active and inactive group, respectively. There was no statistically significant difference in the value of the baseline macular thickness between groups (*P* = 0.663, Mann-Whitney *U* test).

## 4. Discussion

In the literature, we found no publications describing prognostic factors for early morphological therapeutic response to treatment with ranibizumab in patients with wet AMD. We found only articles on prognostic factors for functional therapeutic response. Sarks, Killingsworth, and Gonzales noted that occult CNV may have a good functional treatment response. Sarks et al. [4] and Killingsworth [5] demonstrated histologically that the onset of CNV is characterized by intrachoroidal neovascularization followed by sub-RPE fibrovascular proliferation. Occult CNV which is fibrovascular tissue in the sub-RPE space may in part represent an earlier stage of CNV because it is in the same tissue plane. Up to 50% of occult CNV may then progress to classic CNV. Occult CNV as an earlier stage of the disease is assumed to be associated with less damage to photoreceptors in macula and successful treatment has a better prognosis [6].

In our study, we evaluated the impact of gender, age, baseline BCVA, baseline macular thickness, and type and size of CNV on early morphological therapeutic response following the 3 initial injections of ranibizumab. In the inactive group of 312 patients with complete regression of CNV activity after the initial 3 injections of ranibizumab, we found a statistically significantly higher proportion of occult membranes, statistically significant lower presence of predominantly classic CNV (*P* < 0.001), and statistically significantly lower incidence of CNV > 5 DA (*P* < 0.001) compared with the active group. We observed that smaller and occult CNV lesions have potentially better morphological therapeutic response with the disappearance of the CNV activity and resorption of the macular edema.

We found no significant impact of gender, age, value of baseline BCVA, or baseline macular thickness on early morphological therapeutic response after the initial 3 injections of ranibizumab.

The question remains whether positive morphological therapeutic outcomes are connected to positive functional results. Additional studies are needed to further clarify the relationship of morphological and functional results in ranibizumab treated patients with wet age-related macular degeneration.

## 5. Conclusion

The results showed positive early morphological therapeutic response with restored foveal depression and no signs of exudation on OCT in patients with higher incidence of occult CNV, lower incidence of predominantly classic CNV, and lower incidence of CNV > 5 DA.

There was no evidence of any effect of age, gender, baseline best corrected visual acuity, or baseline macula thickness on the early anatomical restoration of the macula. We believe that occult CNV and a CNV smaller than 5 DA are optimistic for better morphological therapeutic response at the beginning of ranibizumab therapy. To determine if other factors influence the morphological response to ranibizumab treatment in patients with wet AMD, further clinical studies are needed.

## Appendix

Participating AMADEUS Clinical Sites are as follows.

Department of Ophthalmology, Faculty of Medicine in Hradec Králové, Charles University in Prague and University Hospital in Hradec Králové, Czech Republic: Associate Professor Jan Studnička MD., Ph.D., Jaroslava Dusová MD., Ivana Cermanová, Gabriela Blažková; Department of Ophthalmology, First Faculty of Medicine, Charles University in Prague and General University Hospital in Prague, Czech Republic: Zora Dubská, MD., Ph.D., Bohdan Kousal, MD; Department of Ophthalmology, University Hospital, Olomouc, Czech Republic: Professor Jiří Řehák, MD., Ph.D., FEBO, Oldřich Chrapek, MD., Ph.D., Zuzana Prachařová, MD., Martin Šín, MD., Ph.D., FEBO; Department of Ophthalmology, First Faculty of Medicine, Charles University in Prague and Central Military Hospital in Prague, Czech Republic: Jan Ernest, MD., Ph.D; Department of Ophthalmology, University Hospital, Brno, Czech Republic: Associate Professor Petr Kolář, MD., Ph.D., Daniela Vysloužilová, MD., Veronika Matušková, MD; Department of Ophthalmology, Masaryk Hospital, Ústí nad Labem, Czech Republic: Martin Hovorka, MD., Martina Závorková, MD; Department of Ophthalmology, University Hospital, Ostrava, Czech Republic: Jan Němčanský, MD., Pavel Šmehlík, MD; Department of Ophthalmology, Faculty of Medicine in Plzeň, Charles University in Prague and University Hospital in Plzeň, Czech Republic: Dagmar Frdlíková, MD., Hana Fidranská, MD., Tomáš Nathanský, MD; Department of Ophthalmology, Teaching Hospital Královské Vinohrady, Prague, Czech Republic: Miroslav Veith, MD., Stanislava Pokorná, MD.
